# 3D Printing and Performance Study of Porous Artificial Bone Based on HA-ZrO_2_-PVA Composites

**DOI:** 10.3390/ma16031107

**Published:** 2023-01-27

**Authors:** Hongling Bie, Honghao Chen, Lijun Shan, C. Y. Tan, M. S. H. Al-Furjan, S. Ramesh, Youping Gong, Y. F. Liu, R. G. Zhou, Weibo Yang, Honghua Wang

**Affiliations:** 1 Artificial Intelligence Applications College, Shanghai Urban Construction Vocational College, Shanghai 201415, China; 2 School of Mechanical Engineering, Hangzhou Dianzi University, Hangzhou 310018, China; 3 Center of Advanced Manufacturing and Material Processing, Department of Mechanical Engineering, Faculty of Engineering, Universiti Malaya, Kuala Lumpur 50603, Malaysia; 4 State Key Laboratory of Mechanics and Control of Mechanical Structures, Nanjing University of Aeronautics and Astronautics, Nanjing 210016, China; 5 Collaborative Innovation Center of High-End Laser Manufacturing Equipment (National “2011 Plan”), Zhejiang University of Technology, Hangzhou 310023, China; 6 Key Laboratory of E&M, Zhejiang University of Technology, Ministry of Education & Zhejiang Province, Hangzhou 310023, China; 7 Wenzhou Institute of Hangzhou Dianzi University, 3-4/F, Building B, Zhejiang Yungu, Nanyang Avenue, Yaoxi Street, Hangzhou 325038, China; 8 Zhejiang Guanlin Machinery Limited Company, Huzhou 313300, China

**Keywords:** artificial bone, biomanufacturing, finite element analysis, biological scaffold

## Abstract

An ideal artificial bone implant should have similar mechanical properties and biocompatibility to natural bone, as well as an internal structure that facilitates stomatal penetration. In this work, 3D printing was used to fabricate and investigate artificial bone composites based on HA-ZrO_2_-PVA. The composites were proportionally configured using zirconia (ZrO_2_), hydroxyapatite (HA) and polyvinyl alcohol (PVA), where the ZrO_2_ played a toughening role and PVA solution served as a binder. In order to obtain the optimal 3D printing process parameters for the composites, a theoretical model of the extrusion process of the composites was first established, followed by the optimization of various parameters including the spray head internal diameter, extrusion pressure, extrusion speed, and extrusion line width. The results showed that, at the optimum parameters of a spray head diameter of 0.2 mm, extrusion pressure values ranging from 1–3 bar, a line spacing of 0.8–1.5 mm, and a spray head displacement range of 8–10 mm/s, a better structure of biological bone scaffolds could be obtained. The mechanical tests performed on the scaffolds showed that the elastic modulus of the artificial bone scaffolds reached about 174 MPa, which fulfilled the biomechanical requirements of human bone. According to scanning electron microscope observation of the scaffold sample, the porosity of the scaffold sample was close to 65%, which can well promote the growth of chondrocytes and angiogenesis. In addition, c5.18 chondrocytes were used to verify the biocompatibility of the composite materials, and the cell proliferation was increased by 100% when compared with that of the control group. The results showed that the composite has good biocompatibility.

## 1. Introduction

Bone tissue engineering is performed by constructing the three components of artificial bone material, bone cells, and the growth factors required by the cells into a composite and implanting them into the body to reconstruct the tissue externally, internally, and functionally [1]. At present, the main artificial bones on the market are made of metal materials, bioceramics, and polymer materials. Metal materials are widely used in load-bearing bone implants because of their excellent mechanical properties [2,3], including their high tensile strength, high yield strength, high fatigue resistance, and creep resistance. They are frequently used in hip prostheses [4], knee joint prostheses [5], interbody fusion cages [6], etc. The biggest challenge for metal bone implants is their poor bone conductivity, which may shorten their lifespans or even lead to implant failure. In addition, in metal implants, because the differentiation rate of the osteoblasts is much slower than that of calcium phosphate (CaP), it can no longer be absorbed into the metal implants, thus preventing bone remodeling, and is not conducive to the further healthy development of bones. Bioceramic has been widely used in contexts such as artificial tooth roots, tooth back reinforcement [7,8], joint repair [9], heart valve replacement [10], etc. According to the different interactions between bioceramic materials and tissues in vivo, bioceramics can be roughly divided into three categories: (1) inert biomaterials, which do not react or have very little reaction with tissues in vivo, such as alumina, zirconia, carbon, etc.; (2) surfactant materials, which have some chemical interaction with tissue interfaces and can form osseous bonds, such as bioactive glass, hydroxyapatite, etc.; and (3) degradable materials, which will be gradually degraded or absorbed in vivo, and are a kind of temporary bone reconstruction materials, such as gelatin, collagen, chitosan, polylactic acid, and so on. The ideal bone substitute material should have the characteristics of biocompatibility, osteogenesis, structural support, absorbability and degradation, convenient clinical use, and low cost.

Hydroxyapatite (HA) has a molecular formula of Ca_10_(PO_4_)_6_(OH)_2_, a Ca/P ratio of 1.67, a theoretical density of 3.156 g/cm^3^, a refractive index of 1.64–1.65, and a Morse hardness of 5. It has better biological activity than other calcium phosphate ceramics. Stefania De Luca et al. studied the coating of three-dimensional porous glass-derived scaffolds with hyaluronic acid (HA)-fatty acids, which are expected to combine the bone-bonding properties of the glass with the wound-healing promotion carried out by the polymeric conjugates [11]. Miki Hoshi studied collagen (Col)/hydroxyapatite (Hap)/acidic gelatin (AG)/basic fibroblast growth factor (b-FGF) constructs with enhanced bone-forming capability, which might bring about novel bone-forming biomaterials [12]. In addition, compared with other calcium phosphate-based materials, the artificial bone prepared by hydroxyapatite will face multi-energy UV and X-ray detection in the later stage, which may cause the aging of artificial bone [13,14].

HA is also used in 3D printing [15] to make implants with complex structures and is widely used in bone tissue repair and replacement technologies, plastic surgery, and oral restoration, as artificial bone and artificial teeth filling and replacement materials. It has great application value in the field of hard tissue repair materials and is considered to be the most promising substitute material for artificial bone and artificial teeth. However, pure hydroxyapatite material is brittle and has poor mechanical strength, so it is difficult to use it to replace supporting bone, which limits its application in the field of bone tissue repair. Adding a certain amount of a toughening material such as alumina and zirconia [16,17,18,19,20] to hydroxyapatite can improve the mechanical properties of the resulting body. Stabilized zirconia is often used as a toughening material in the preparation of hydroxyapatite ceramics. It can significantly improve the hardness, bending strength, and fracture toughness of hydroxyapatite composite ceramics. Good bone repair materials should not only have a high mechanical strength but also have connected micropore structures similar to those of human bones. Related research results show that porous hydroxyapatite bone repair materials with suitable numbers and sizes of pores can provide growth space for osteoblasts and blood vessels and promote rapid bone growth [21,22,23,24,25,26,27]. Porous hydroxyapatite bone ceramics have good osteogenic induction properties and enhance the capacity for bone defect repair, which is more in line with the clinical requirements for biological bone repair materials.

Although research results for porous artificial bone based on zirconia reinforced hydroxyapatite have been reported, the research on how different proportions of zirconia affect the fluid properties of the composite slurry, how to optimize the specific parameters of 3D printing according to the rheological properties of the slurry, and the biocompatibility of various component samples has yet to be performed. In this work, the printing scheme for a new composite composed of hydroxyapatite-zirconia-polyvinyl alcohol porous artificial bone was studied using the 3D printing equipment 3D-BIOPLOTER. By configuring the new composite material, studying the characteristics of the new composite material, and analyzing the hydrodynamics of the new composite material, a theoretical model of the composite extrusion process is established. Through a numerical simulation using the software FLUENT, the optimal printing parameters were obtained, and they were then verified through an experimental process. Finally, the mechanical properties and biocompatibility of the sintered scaffolds were tested to further verify the feasibility of the composites as human implants.

## 2. Materials and Methods

### 2.1. Material Composition

In bone tissue engineering, the preparation of artificial bone implants with excellent mechanical properties and good biocompatibility is the focus of current research in the field of artificial bone. Artificial bone implants need to assume the support of the defective area after implantation into the organism and, therefore, need to have strong mechanical properties. Hydroxyapatite ceramics have naturally excellent biocompatibility and osteoinductivity, but an important drawback is their poor mechanical properties, so a single hydroxyapatite ceramic cannot be used as a bone repair material for the fabrication of artificial bone implants. Currently, a more feasible approach is to meet the need for both the mechanical properties and the biocompatibility of artificial bone materials by adding a composite material with excellent mechanical properties. Therefore, a biologically inert material with good elasticity, toughness, and stability can be used in composite formulations with hydroxyapatite material to combine the advantages and strengths of both materials and obtain a composite material that meets the requirements for bone implants. ZrO_2_ is chemically inactive and has a high melting point, a low coefficient of thermal expansion, and mechanical properties that meet the needs of artificial bone toughening. Since the composite material in the powder state cannot be extruded by pneumatic 3D printing equipment, the powder needs to be mixed with a liquid binder to form a viscous slurry to facilitate the extrusion of the artificial bone composite material through the equipment. The artificial bone pellet thus formed is then post-processed to break down the binder and obtain artificial bone containing only hydroxyapatite and ZrO_2_. Polyvinyl alcohol is a cell culture fluid used in recent scientific experiments, including a new finding that validates its good biocompatibility. In addition, its aqueous gels are widely used in ophthalmology, wound dressings, and artificial joints [28,29,30], and, in this work, polyvinyl alcohol type PVA1788 was chosen as the binder.

### 2.2. Material Composition

The composite pastes for injection molding type 3D printing devices need to meet certain conditions:

(1) The material itself has the right viscosity to maintain its shape after extrusion.
(2) There is a uniform mixing of materials, capable of stable extrusion.
(3) The material itself is in a stable state and does not change its chemical or physical properties over time.

**Figure 1:**
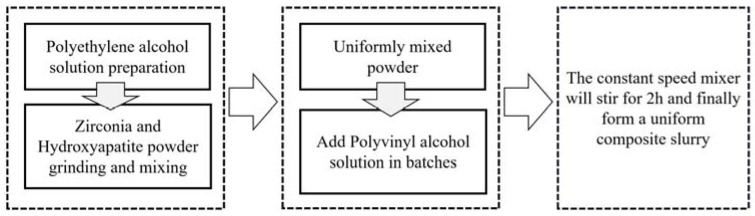
Preparation process of composite slurry.

The solid phase content in the composite slurry has a great impact on the molding quality. When the solid phase content in the composite slurry is too high, the slurry’s viscosity is too great, and the extrusion process is prone to clogging the nozzle and causing breakage; when the content is too low, the composite slurry’s viscosity is too low to maintain a certain shape. This paper uses the PVA1788 type polyvinyl alcohol (purchased from Aladdin, Shanghai, China). After a large number of experiments measuring PVA1788 type polyvinyl alcohol in a 14% concentration, we know that its viscosity is stable and will not change over time. Therefore, the concentration of polyvinyl alcohol chosen in this paper is 14%. The specific dissolution steps can be shown in Figure 1.

(1) Add the appropriate amount of polyvinyl alcohol powder slowly to deionized water at room temperature several times under the stirring of a constant speed mixer to make it disperse evenly, and keep stirring for 30 min.
(2) Put the beaker with polyvinyl alcohol which is well dispersed in a digital thermostat water bath with the temperature set at 75 °C. As the water temperature rises, continue to stir for 1~2 h until the polyvinyl alcohol is fully dissolved.
(3) Leave the configured polyvinyl alcohol solution to defoam and then place it in a wide-mouth bottle in a reagent box for storage until use.
(4) Weigh a proper amount of zirconia powder (AR, purchased from Aladdin company, China) and hydroxyapatite powder (AR, purchased from Aladdin company, China), respectively, and then place them in the planetary mixer for grinding and mixing for 1 h to make them disperse evenly. The particle size of hydroxyapatite powder is mainly 18–23 μm, while that of zirconia powder is mainly 17–22 μm.
(5) Use a disposable dropper to suck a proper amount of polyvinyl alcohol solution from the jar and drop it into the beaker, and use magnetic stirrer (Ika, (Staufen, Germany)) to stir it evenly.

### 2.3. Printing Equipment and Test Equipment

**Figure 2:**
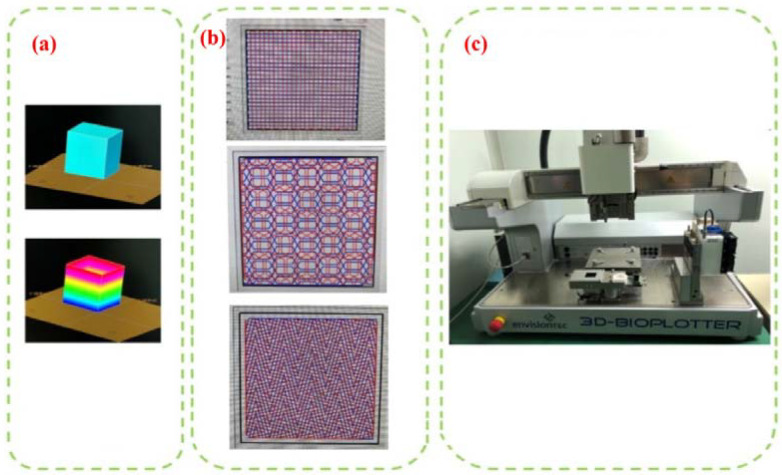
3D model pre-processing: (**a**) Slice processing; (**b**) Internal structure selection; (**c**) 3D-bioplotter.

In this paper, 3D-BIOPLOTTER printing (Envisiontec, Gladbeck, Germany) is used to prepare the scaffold, which is a kind of rapid prototyping equipment that can use a variety of biomaterials, as shown in Figure 2c. The biological scaffold manufactured by it has an external form and an open internal structure that meet the design requirements.

(1) *Slice Processing*

Slicing is the process of transcoding data files (.stl, .obj, etc.) into printing device action data (gcode), which can also be understood as dividing an entity into several identical layers, where the divided layers are the path of the 3D printing process, as shown in Figure 2a. The most important thing in the slicing process is the layer thickness, that is, the thickness of each piece. If the layer thickness is smaller, the relative accuracy of the overall model will be higher, and the surface texture will be better. Surface texture refers to the fact that 3D printing creates layers of molding marks on the surface due to layered manufacturing. According to the available information, the layered thickness is generally 80% of the print nozzle diameter.

(2) *Internal Structure Selection*

The internal structure of the bio-scaffold must be interconnected due to the environment in which the bio-scaffold is used so that the cells attached to the bio-scaffold can take in beneficial substances and excrete waste through channels when the bio-scaffold is functioning in the organism. Different internal structure types can be selected through the corresponding module of the software. Different structure types are subject to different forces due to the different areas of bonding between the lines of the printed and formed materials. The usual internal filling forms are linear, folding, and honeycomb, as shown in Figure 2b.

### 2.4. Optimization of Printing Parameters Based on Theoretical Modeling and Simulation of Composite Extrusion Process

In this paper, theoretical modeling and simulation of the composite extrusion process are carried out to achieve the parameter optimization of the stent printing process. Firstly, a model of the composite slurry flow in the extrusion process of the pneumatic extrusion 3D printing equipment is established, the flow characteristics of composite slurry and the theoretical equations of the main factors affecting the molding accuracy (extrusion flow rate, extrusion speed, line width, porosity) are analyzed, and the simulation of the composite slurry flow process is carried out using ANSYS FLUENT software to obtain the effects of different air pressures and different nozzle diameters on the extrusion volume. The effects of different air pressures and different nozzle diameters on the extrusion volume were successfully obtained, which provides a reference for the subsequent printing of the support structure [31,32].

#### 2.4.1. Theoretical Model Construction of Composite Material Extrusion

**Figure 3:**
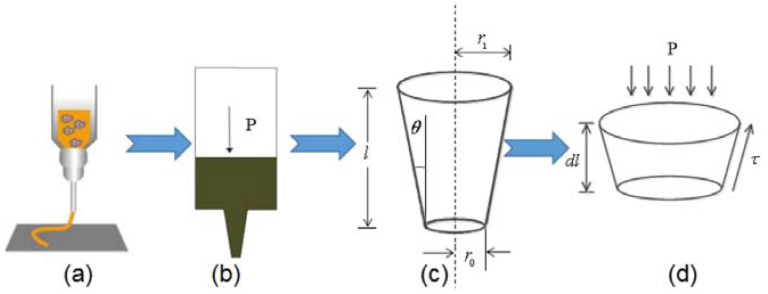
The simplified print head of bioplotter: (**a**) Extrusion process of composite materials; (**b**) Simplified model of composite extrusion process; (**c**) Print nozzle end model; (**d**) Shear force unit model of printing nozzle.

The print head of the bioplotter can be simplified as shown in Figure 3. Sodium alginate gelatin solution is stored in advance in the syringe, as shown in Figure 3a For a printhead of unit length, the equation of force balance between the material and the printhead contact surface at unit extrusion pressure can be written as Equation (1).

(1) $$\pi r^{2}dp=2\pi r.\tau \tan\theta dl$$

where *r* is the radius of needle; *p* is the pressure; *θ* is the half cone angle, and *l* is the length of the tapered needle, while *r*_1_ and *r_o_* are, respectively, the inlet and outlet radii of the needle head.

The relationship between the strain rate and the shear flow is Equation (2).

(2) $$\gamma =\frac{4Q}{\pi r^{3}}$$

where *γ* is the shear speed; *r* is the nozzle exit radius; *Q* is the total flow.

According to the power law fluid model, we can obtain the fluid shear stress, as shown in Equation (3).

(3) $$\tau =k{\left(\frac{4Q}{\pi r^{3}}\right)}^{n}$$

where *τ* is the shear stress; *n* is the flow coefficient; *K* is the composite consistency factor.

For a defined printhead, the tangent of the angle can be expressed as Equation (4).

(4) $$\tan\theta =\frac{dr}{dl}$$

where *l* is the conical needle length and *r* is the conical needle exit radius.

Combining Equations (1)–(4), the expression for the input air pressure *P* can be expressed as Equation (5).

(5) $$p=\int_{r}^{R}\frac{2\pi r\tau \sec\theta \cos\theta }{\pi r^{2}\tan\theta }\;dr=\frac{2k}{3n\tan\theta }{\left(\frac{4Q}{\pi r^{3}}\right)}^{n}\left[1-{\left(\frac{r}{R}\right)}^{3n}\right]$$

The total flow rate extruded from the printhead can be expressed as Equation (6).

(6) $$Q=\frac{\pi r^{3}R^{3}}{4}{\left[\frac{3nP\tan\theta }{2K\left(R^{3n}-r^{3n}\right)}\right]}^{\frac{1}{n}}+\frac{\pi hr^{2}R^{3}}{k^{\frac{1}{n}}}\left[\frac{3nP\tan\theta }{2\left(R^{3n}-r^{3n}\right)}\right]$$

The extrusion speed can be expressed as Equation (7).

(7) $$\nu =\frac{\pi rR^{3}}{4}{\left[\frac{3nP\tan\theta }{2K\left(R^{3n}-r^{3n}\right)}\right]}^{\frac{1}{n}}+\frac{\pi hr^{2}R^{3}}{k^{\frac{1}{n}}}{\left[\frac{3nP\tan\theta }{2\left(R^{3n}-r^{3n}\right)}\right]}^{\frac{1}{n}}$$

where *R* is the nozzle inlet radius; *P* is the given pressure value; *h* is the slip layer thickness; and *r* is the nozzle outlet radius. The line width of the extruded line can be expressed as Equation (8).

(8) $$w=\frac{Q}{h\nu^{\prime }}-\frac{\pi h}{4}=\frac{\frac{\pi r^{3}R^{3}}{4}{\left(\frac{3nP\tan\theta }{2K\left(R^{3n}-r^{3n}\right)}\right)}^{\frac{1}{n}}+\frac{\pi hr^{3}R^{3}}{k^{\frac{1}{n}}}{\left(\frac{3nP\tan\theta }{2\left(R^{3n}-r^{3n}\right)}\right)}^{\frac{1}{n}}}{h\nu^{\prime }}-\frac{\pi h}{4}\;$$

From Equation (8), the influencing factors of the line width include the layer height, nozzle moving speed, extrusion pressure, nozzle inner diameter, material consistency, etc. In each printing process, the layer height is determined according to the size of the printing nozzle, and the material consistency, nozzle diameter, etc. are also relatively determined.

The scaffold model was constructed by stacks of highly identical multilayered structures, and the scaffold can thus be viewed as a whole divided into several unit architectures. Thus, if one were to calculate the proportion of the material fraction within each unit over the entire unit, one could calculate both the material free fraction and, in turn, the stomatal rate of the overall model. Depending on the dimensions of the cell structure, the theoretical porosity of the bone scaffold *δ* can be expressed as Equation (9):

(9) $$\delta =1-\frac{\frac{\pi r^{3}R^{3}}{4}{\left(\frac{3nP\tan\theta }{2K\left(R^{3n}-r^{3n}\right)}\right)}^{\frac{1}{n}}+\frac{\pi hr^{3}R^{3}}{k^{\frac{1}{n}}}{\left(\frac{3nP\tan\theta }{2\left(R^{3n}-r^{3n}\right)}\right)}^{\frac{1}{n}}}{h\nu^{\prime }L}-\frac{\pi h}{4}-\frac{\pi h}{4L}$$

#### 2.4.2. Finite Element Simulation of Extrusion Process

In this section, we simulate the extrusion process of the composite material in the silo using ANSYS FLUENT. The extrusion process is as follows: a certain pressure is applied at the air pressure inlet at the top of the silo, and the composite material fills the silo. The boundary conditions are both the pressure inlet and the pressure outlet, and some assumptions about the flow process need to be made before the extrusion process simulation with FLUENT because, as the composite material is a complex fluid, its flow process in the silo is influenced by gravity, friction, and material viscosity. Therefore, in order to speed up the calculation and make the simulation process realistic, the following assumptions were made [33].

(1) The flow of the composite material in the silo is incompressible.
(2) The influence of gravity is ignored.
(3) The pressure drop caused by the non-pressure setting value is ignored.

**Figure 4:**
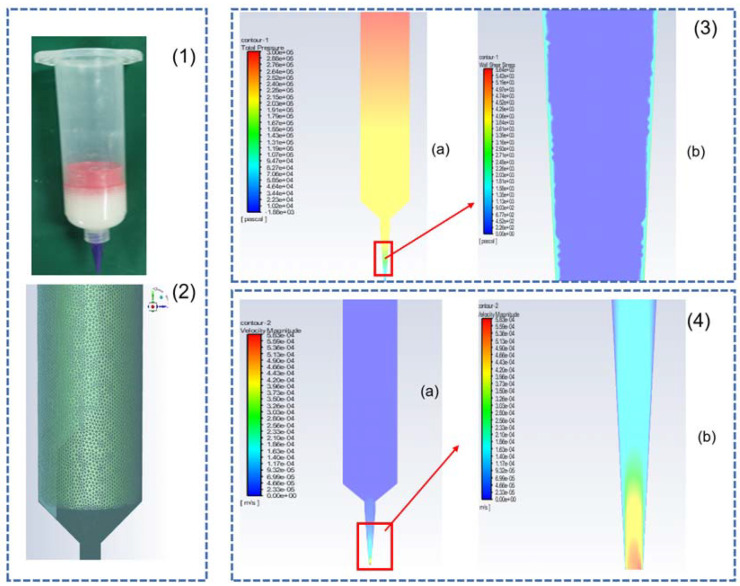
Extrusion process model construction and simulation results: (**1**) The original structure of the extrusion bin; (**2**) The mesh model of the extrusion bin; (**3**) The pressure gradient distribution from: (**a**) Pressure distribution; (**b**) The magnifications of the print nozzle’s internal shear force distribution; (**4**) The change of the material flow rate at different positions: (**a**) the change of the material flow rate Overall drawing; (**b**) The magnified view of the flow rate variation at the print nozzle position.

Figure 4(1) shows the original structure of the extrusion bin, and Figure 4(2) shows the mesh model of the extrusion bin. The mesh division is the process of discretizing the solution as a whole into accurately solvable cells, and the quality of the mesh has a decisive influence on the solution results.

Figure 4(3) shows the pressure gradient distribution from the air pressure inlet to the material outlet when the composite material is extruded from the bin at an inlet air pressure of 3 bar and with a nozzle inner diameter of 400 microns. From Figure 4(3a), it can be seen that the maximum value of the air pressure at the top of the bin, from the top of the bin to the nozzle outlet pressure valve, gradually decreases, reaching a minimum value at the nozzle outlet. From Figure 4(3b), which shows the magnifications of the print nozzle’s internal shear force distribution in the tube wall, it can be found that the shear force changes with different reducers. When the shear force reaches the critical value, the material will exhibit a slip phenomenon. Figure 4(4a) shows the change of the material flow rate at different positions during the extrusion process. The deepening of the color represents the acceleration of the flow rate, and it can be seen that the flow rate of the material in the bin does not change much. When the composite material enters the print nozzle, the flow rate accelerates with the change in the inner diameter of the print nozzle. This shows that the assumptions made earlier in this chapter are reasonable. Figure 4(4b) shows a magnified view of the flow rate variation at the print nozzle position, which shows that the flow rate at the tip of the print nozzle reaches the maximum value of the flow rate for the whole extrusion process. In order to study the effects of air pressure and nozzle diameter on the extrusion flow rate, using a model in which other conditions remain unchanged, change the boundary conditions—the pressure value at the entrance and the exit diameter, respectively—to derive different extrusion velocities. The corresponding flow rate values are obtained using Equation (6).

**Figure 5:**
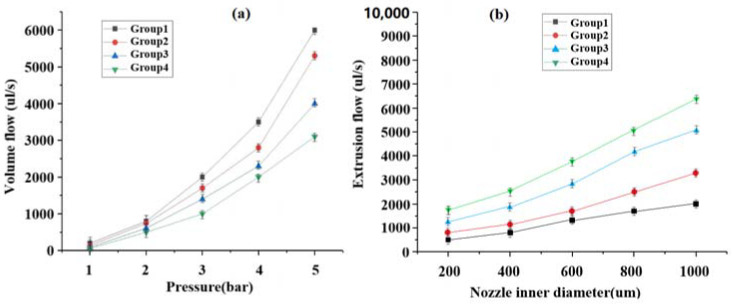
The extrusion flow rate correlated with the air pressure and the inner diameter of the nozzle: (**a**) Extrusion flow rates at different pressures; (**b**) Extrusion flow rates for the different inner diameters.

The air pressure values were set to 1 bar, 2 bar, 3 bar, 4 bar, and 5 bar, and the extrusion flow rates at different pressures were shown in Figure 5a. The nozzle diameters were set to 200 µm, 400 µm, 600 µm, 800 µm, and 1000 µm, and the extrusion flow rates for the different inner diameters are plotted as shown in Figure 5b.

From Figure 5a,b, it can be seen that the extrusion flow rate is positively correlated with the air pressure and the inner diameter of the nozzle, and that the extrusion flow rate change multiplier is larger when the air pressure value is larger; in addition, the change of the nozzle’s inner diameter value has a relatively small effect on the extrusion flow rate change multiplier, which indicates that the air pressure is the key factor affecting the extrusion flow rate.

## 3. Results and Discussion

During the artificial bone-forming process, the composite materials are stacked layer by layer according to the data and settings pre-cut by the computer software, and each layer is composed of lines of different structures and different thicknesses, so the material content of each line determines the forming thickness of the layer, and too much or too little extruded material will result in a deviation from the pre-designed dimensions. In the manufacturing process for pneumatic injection molding additive devices, air pressure is the only source of power used to extrude the composite material from the bin onto the print substrate, and the magnitude of the air pressure severely affects the flow rate of the extruded composite material, so it is important to study the effect of air pressure in the artificial bone molding process.

### 3.1. Viscosity Test of Different Slurries

Most biological fluids and high concentration solutions of polymer are non-Newtonian fluids. Non-Newtonian fluids can be divided into time-varying and non-time-varying non-Newtonian fluids according to the relationship between the viscosity function and the shear duration. According to different conditions of use, different non-Newtonian fluids have different rheological equations. In this study, hydroxyapatite powder and zirconia powder were mixed and bonded with polyvinyl alcohol solution, which belongs to a glue model for which the power law equation is applicable in theory. The viscosity model can thus be expressed as Equation (10).

(10) $$\tau =K{\left(\frac{du}{dy}\right)}^{n}$$

where *K* is the viscosity coefficient, which is taken according to the property of the fluid, and *n* is the fluidity index, representing the degree of deviation from Newtonian fluid. The viscosity of the final configured composite slurry differs according to the percentage of total solids accounted for by the hydroxyapatite powder and the ZrO_2_ powder, and four different groups of ratios were set according to ZrO_2_ contents of 0%, 10%, 20%, and 30%, as shown in Table 1.

**Figure 6:**
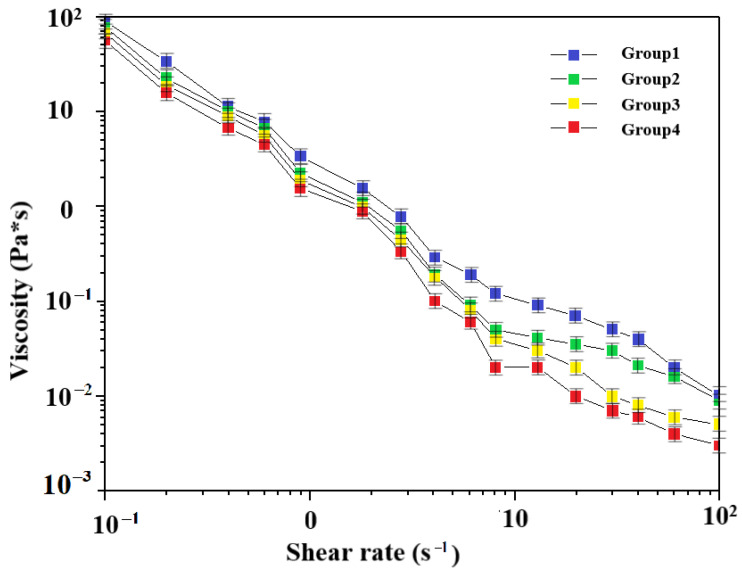
Results of viscosity characteristics of the composite slurry.

The viscosity of the composite slurry made under different shear rates was measured using a viscometer for each group of ratios, and the viscosity characteristics of the composite slurry made under different ratios are shown in Figure 6.

By measuring the viscosity of the composite slurry at each group ratio, it was found that the viscosity of each composite group was inversely proportional to the viscometer speed; the viscosities of the composite slurries configured with different ZrO_2_ percentages of the composite powder were slightly different; and, the higher the ZrO_2_ content at the same viscosity, the greater the shear rate of the composite slurry [34]. For the pneumatic injection molding equipment, the extrusion force comes from the air pressure provided by the external supply equipment. Therefore, it is very important to choose the compound slurry with the actual viscosity from the molding process.

### 3.2. Printed Parameter Air Pressure and Flow Rate Relationship Verification

**Figure 7:**
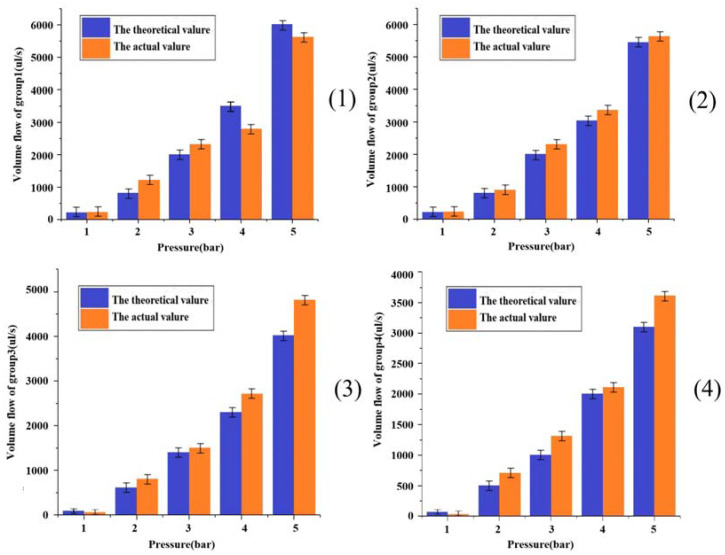
Results for extruded material flow rate at different air pressures: (**1**) Theoretical value and actual measured value of flow rate of group1 of materials under different pressures; (**2**) Theoretical value and actual measured value of flow rate of group2 of mate-rials under different pressures; (**3**) Theoretical value and actual measured value of flow rate of group3 of materials under different pressures; (**4**) Theoretical value and actual measured value of flow rate of group4 of materials under different pressures.

The composite materials with the material ratio and grouping configuration listed in Table 2 shall be tested to determine the flow rates of the extruded materials under different air pressures. The nozzle size selected for the test is 400 µm, the air pressure of the conical nozzle is set according to 1 bar, 2 bar, 3 bar, 4 bar, and 5 bar, respectively, as shown in Table 2, and the results are shown in Figure 7.

It is known from Figure 7 that the flow rate of the extruded material increases exponentially with increases in pressure, and that, as the pressure continues to increase, the values of the extruded flow rate show a significantly increasing trend. The different increasing trend illustrates that the flow coefficient of the composites is not equal to one, as according to the formula through which the fluid type is judged by the flow coefficient, which verifies the discrimination of the fluid type of the composites. From the analysis of Figure 7, it is found that for the extrusion flow under different ratio grouping, the overall trend increases with the increase of gas pressure. At the same ratio, the actual and theoretical extrusion flow rates at different gas pressures are also different. In the two cases with larger gas pressure versus viscosity, the theoretical versus actual values had relatively large errors, and this phenomenon could be verified from the theoretical versus real control of group 3 versus group 4 at 5 bar of pressure. This deviation of actual values from theoretical values arises from a variety of factors, and from a gas pressure perspective, there may be stochastic cases of the instability of the air pressure at the extrusion pressure of the device; in terms of temperature, an increase in device run time that includes an increase in the printing ambient temperature can cause the actual temperature to differ from the theoretically calculated one. Overall, in the first group of allocation, the maximum value of error was 7.6% and the minimum value was 5.3%. The difference between the allocation medium and the error of the second group was 6.3% at its largest and 2.1% at its smallest; the margin of error was 8% at maximum and 1.7% at minimum in group 3; and the maximum value of the error was 9.1% while the minimum value was 4.2% in group 4. The maximum error did not exceed 10% in any group. From the point of view that it is not possible to achieve complete consistency between the actual operating procedure and the ideal condition, an error of 10% is within the allowable range [35].

### 3.3. Effect of Internal Diameter Extrusion Pressure from Different Spray Heads on Extrusion Speed

The extrusion speed, since it cannot be obtained by direct measurement, can be obtained indirectly by weighing the mass of the extruded composite material per unit of time, according to Equation (11).

(11) ν=Qt

where *Q* is the extrusion flow rate and *t* is the extrusion time.

The extrusion mass can be expressed as Equation (12).

(12) m=ρV

where *m* is the mass of the composite material and *ρ* is the average density of the composite material. This combined with the extrusion speed Equation (8) can be rendered as Equation (13).

(13) $$m=\rho \nu t.\pi r^{2}$$

**Figure 8:**
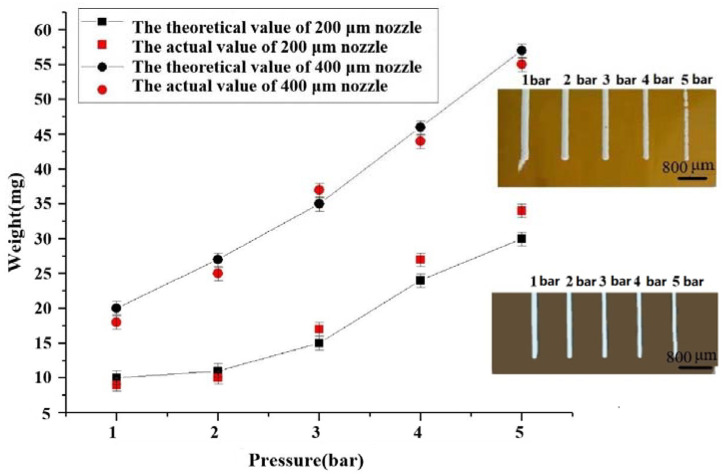
Variation of extruded material mass at different air pressures for two nozzle diameters.

In order to ensure a linear relationship between mass and extrusion speed, the remaining variables need to be set to constant values. From the extrusion speed Equation (9), it can be seen in the extrusion speed and the extrusion flow rate are positively correlated. According to Equation (12), the additional factors affecting the extrusion speed are the flow rate and the cross-sectional area of this variable, which for this study that is the nozzle diameter. Studying the changes in the extrusion flow rate for different nozzle diameters at different air pressures is thus very necessary to verify the rationality of the theoretical formula, so the test parameters are set as follows. The air pressure is set as five different groups of pressure values: 1 bar, 2 bar, 3 bar, 4 bar, and 5 bar; the nozzles have diameters of 200 microns, and 400 microns, respectively, and are conical; the experimental material was selected from the second group of proportional data made of composite slurry. The experimental process is as follows: the printhead is adjusted to a safe position, and the weight of a 50 mL beaker is measured using an electronic scale. The 50 mL beaker is used to collect the composite material extruded from the printhead within 30 s, and then placed on an electronic scale to weigh the results. When the weight of the beaker is subtracted, the result obtained is the weight of the extruded composite material, and the effect of the extrusion process is shown in the inset figure in Figure 8.

From Figure 8, it can be seen that the actual extruded mass per unit time at both nozzle diameters differs from the theoretical mass, and the gap value increases with increasing air pressure. At a nozzle inner diameter of 200 μm, the actual extrusion flow rate starts to be greater than the theoretical value of the extruded material mass at the 3 bar input air pressure. This is because the composite material is a pseudoplastic fluid, and its volume will change to a certain extent with the change in pressure. When the value of the air pressure is larger, the input air flow rate per unit time in the bin is greater than the amount of extruded material, resulting in a rise in the pressure at which the composite material is extruded, which causes the flow rate to exceed the theoretical flow rate so that the volume of extruded material per unit time increases. From the longitudinal analysis in the figure, it can be seen that, if we increase the inner diameter of the nozzle at this time, more material will be extruded per unit of time, and the air pressure difference between the air pressure input port and the nozzle in the silo will gradually decrease and finally reach the steady state, so that the actual extruded volume will be smaller than the theoretical volume again. This phenomenon confirms that, in order to achieve a stable extrusion state for composites of different viscosities due to different ratios, not only can the input air pressure be adjusted, but the cross-sectional area of the flow can also be changed by replacing the nozzle, thus changing the extrusion volume [36].

### 3.4. The Effect of Different Air Pressure Nozzle Shift Speeds on the Line Width

One is the speed of the extrusion of the material from the printhead, which is proportionate to the extrusion pressure and the internal diameter of the printhead according to the analysis in the previous section; the other is the speed of the movement of the printhead, which is controlled by a pre-inputted value in the computer. If the printhead movement speed is too small, less than the speed of the extrusion of composite materials from the bin, the material deposited on the print substrate per unit time is larger, and thus the actual line width will be larger than the theoretical line width. If the printhead movement speed is greater than the extrusion speed of the composite materials, the flow of the composite materials deposited per unit time is smaller, so that the thickness of each layer of the structure is much smaller than the theoretical thickness, and the overall size of the artificial bone will also deviate from the design size after the layers are stacked [37].

**Figure 9:**
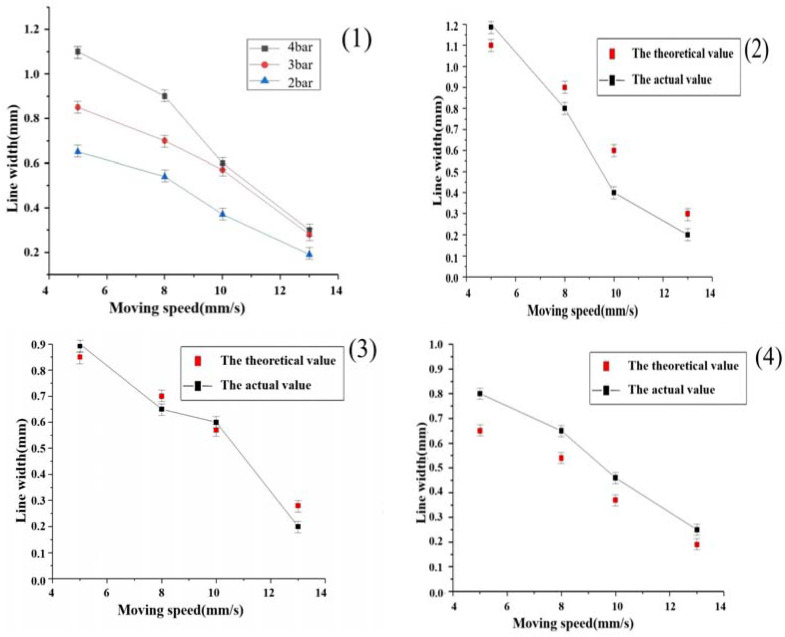
Printhead movement speed on the impact of print line width: (**1**) different pressure printhead movement speed—line width overall trend graph; (**2**) 4 bar pressure under theoretical—actual line width comparison graph; (**3**) 3 bar pressure under theoretical—actual line width comparison graph; (**4**) 2 bar pressure under theoretical—actual line width comparison graph.

From the line width theoretical model in Equation (8), it can be seen that, at the actual print start, the material viscosity characteristics, nozzle diameter, nozzle length, and extrusion speed are constant, so the main factor affecting the line width is the printing nozzle movement speed. In order to verify the reasonableness of the theoretical formula for the line width, according to the actual printing with parameter changes, we set different shift speeds and different air pressure values for multiple groups of experiments. The experimental specific parameter values were set for the experimental material selection of the second group of proportional data, the print nozzle shift speeds were set to 5 mm/s, 8 mm/s, 10 mm/s, and 13 mm/s; the air pressure was set to 2 bar, 3 bar and 4 bar; a nozzle diameter was selected; and we selected the printhead movement speed. The printhead diameter is a 400-micron conical needle, and the slice thickness is set to 0.32 mm. In order to facilitate the observation of the line printing condition, we selected the horizontal and vertical linear internal structure, the line interval X-axis was set to 1 mm and the Y-axis was set to 1.5 mm. The theoretical values were compared with the actual measured values, and the results are shown in Figure 9.

### 3.5. Mechanical Properties Testing of Molded Brackets

**Figure 10:**
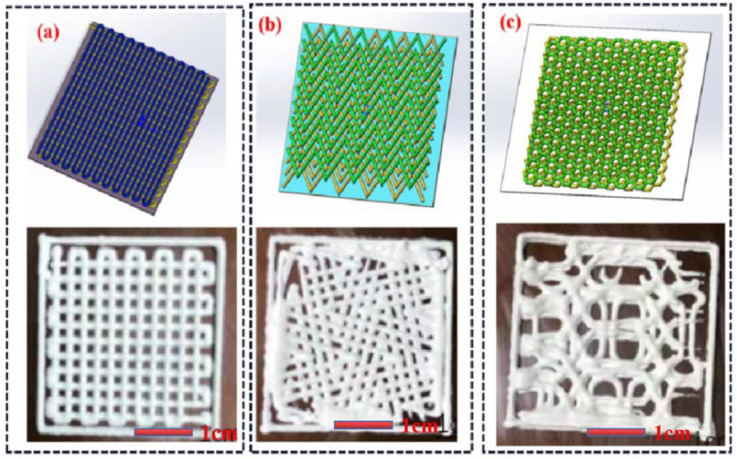
Different internal structures model and printed samples: (**a**) linear schematic; (**b**) bending line schematic; (**c**) honeycomb.

In addition to the physical properties of the material itself, the various internal structures of the artificial bone solids also have different properties that affect the compressive properties of the artificial bone. Before the performance test, in order to further investigate the factors affecting the performance of the artificial bone after molding, the relationship between the internal structure and the mechanical properties was investigated by setting up different internal structures and obtaining the final mechanical property data. In this paper, three different internal structure models were used, as shown in Figure 10. Figure 10a is the linear type, Figure 10b is the bending line type, and Figure 10c is the honeycomb type. Three different internal structures, including the linear type, honeycomb type, and polygonal type, are used for support preparation. The configuration proportion of the composite materials is based on the grouping proportion set in Table 1. The printing nozzle is a cone needle with a diameter of 400 microns. The layer height is set as 0.32 mm, the line spacing is set as 1.1 mm, the moving speed of the nozzle is set as 13 mm/s, and the model size is 10 × 10 × 5 mm. The One-Step sintering (OSS) method was performed by sintering at a target temperature of 1080 °C with a heating rate of 5 °C/min in an air atmosphere using similar heating schemes, and the material was held at the target temperature for 3 h, followed by cooling to 450 °C at a rate of 5 °C/min, and finally cooled to room temperature naturally. The compressive strength of the obtained sample is tested through the electronic universal material testing machine (Universal testing machine, Instron 5566, Shanghai, China), the loading speed is set to 1 mm/min, three experiments are conducted for each group, and the data is taken as the average. The compressive strength is computed using Equation (14) using a universal testing machine operated at a loading speed of 1 mm/min.

(14) $$\sigma =\frac{P}{A}$$

where *σ* is the compressive strength of the specimen, *P* is the load at fracture, and *A* is the area of the specimen profile.

**Figure 11:**
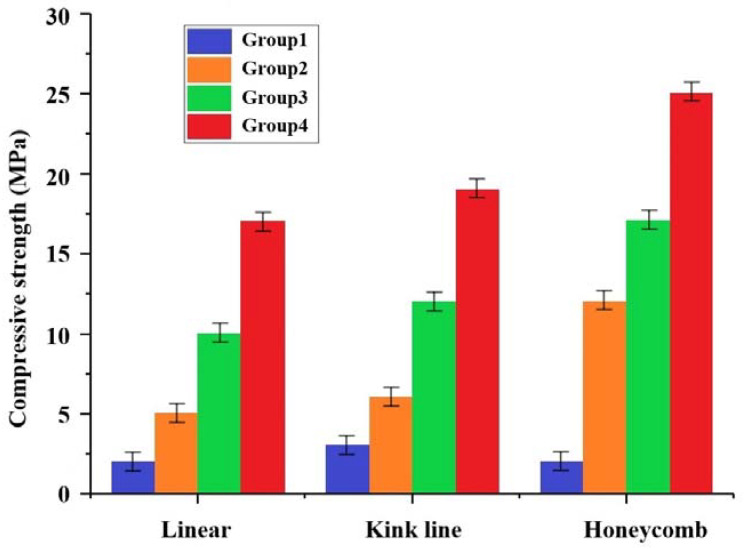
Compressive strength of the artificial bone scaffold in all pore structures.

As shown in Figure 11, the overall compressive strength and modulus of elasticity of the artificial bone scaffold increased with the increase of ZrO_2_ content in each grouping ratio for the same internal structure type; this indicates that the toughening effect of ZrO_2_ in the composite composition is very significant, and the increase of ZrO_2_ content increases the compressive strength of the artificial bone scaffold in all pore structures, and in the linear structure. The compressive strength of the linear structure reached up to 17 ± 1 MPa, and, by changing the internal structure, the compressive strength of the honeycomb type could reach 25 ± 1 MPa under the same ratio, obtaining a performance improvement of nearly 50%, an improvement of nearly two orders of magnitude compared to the compressive strength (<0.5 MPa) of the artificial bone scaffold made of bioceramics using the traditional manufacturing method [38].

**Figure 12:**
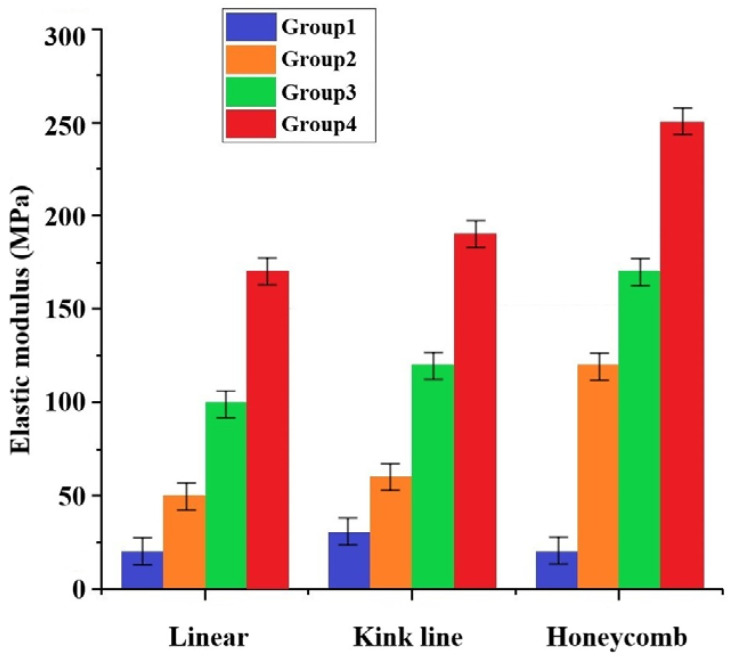
Results of elastic modulus increasing with zirconia for different internal structures.

With the increase in ZrO_2_ content, the elastic modulus of the scaffold obtained different improvement effects, as shown in Figure 12. In the linear type, the elastic modulus of groups 2, 3, and 4 increased by one to five times compared to that of group 1. Different internal structures can obtain different elastic moduli, and the maximum elastic modulus of the honeycomb group can reach 250 MPa. Human bones are divided into cortical and cancellous bones, and different parts have different compressive strengths. The compressive strength of cortical bones generally ranges from 100 MPa to 150 MPa, while the compressive strength of cancellous bones varies from 2 MPa to 20 MPa depending on the density. Therefore, based on the compressive strength alone, the final bone formation in this study can be used as a repair for bone defects in non-load-bearing areas of the human body [39,40]. In clinical applications, different internal structures can be selected to improve the artificial bone performance according to the requirements of different parts of the artificial bone.

**Figure 13:**
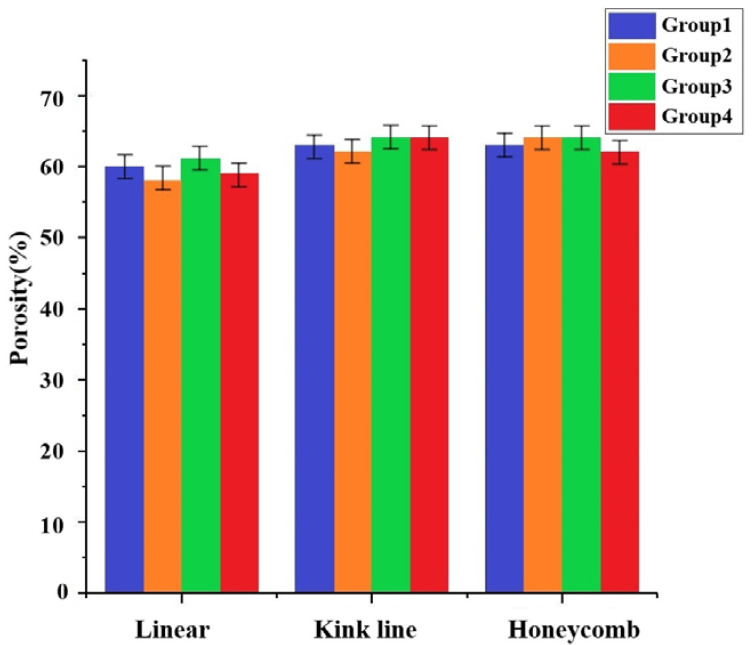
Porosity of zirconia samples with different internal structures.

**Figure 14:**
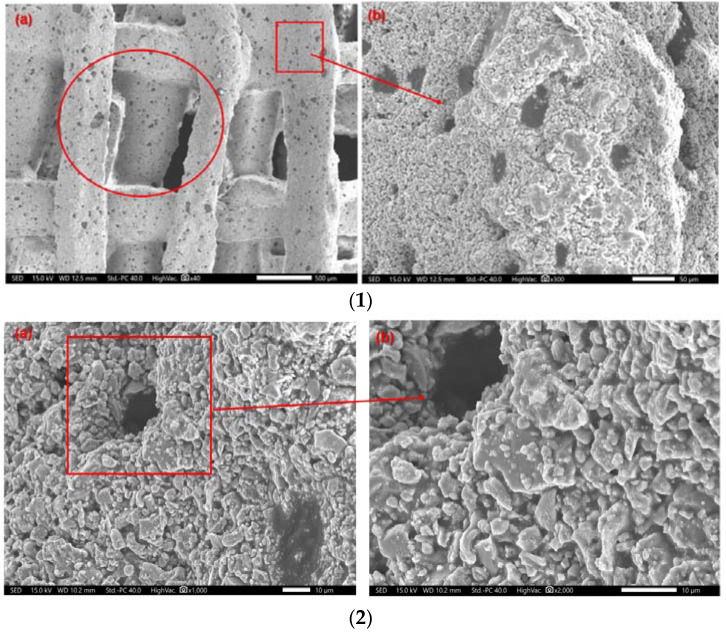

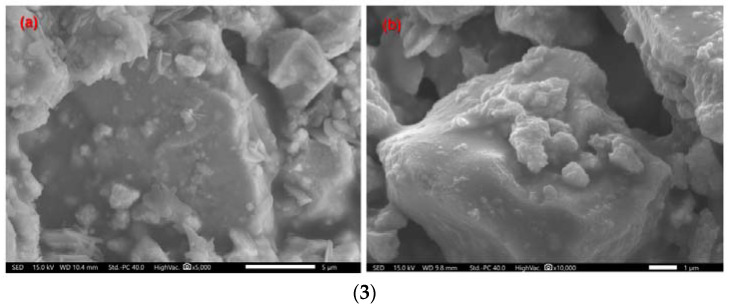
SEM of porous structure of composite scaffolds: (**1**) Scaffold unit and pore distribution; (**2**) Pore structure magnification 1000× HA and zirconia distribution maps; (**3**) Pore structure internal details: (**a**) SEM result after a magnification of 5000× *g*; (**b**) SEM result after a magnification of 10,000× *g*.

The good porosity is helpful for cell culture and blood vessel growth and is also an important measure of bone scaffolds [41]. Figure 13 showed the porosity of scaffold samples with different internal structures. The porosity of the three internal structures of printed stent samples with different levels of zirconia is close to 60%, among which the broken line type sample was the highest, close to 65%. The scaffold voids of the whole composite consist of two kinds, which were tested using scanning electron microscope (SEM, COXEM EM-30+, Korea). The parameters used in scanning were: acceleration voltage 15.00 KV; working distance 9.8 mm; and 40 times, 300 times, 1000 times, 2000 times, 5000 times, and 10,000 times magnification. As shown in the section of Figure 14(1a) represented by circles, and the other pores formed on the structure bulk after sintering as shown in the Figure 14(1a) section represented by boxes, the length, width, and height of the box hole are about 1 mm × 0.75 mm × 0.55 mm; Figure 14(1b) is the pore enlargement results. It can be seen that small pores of various sizes were formed after the sintering of the extrusion bulk structure, and that the hole wall is covered with small holes with a diameter of about 25 microns, resulting in a slight increase in the overall porosity. In Figure 14(2), which shows the specific structure of the pores of the bulk structure, the composite rough interface formed by the larger particles of hydroxyapatite and the smaller particles of zirconia, which helped the adhesion and proliferation of the subsequent cells, can be seen. Figure 14(3a) is the SEM result after a magnification of 5000× *g*, giving a further clear understanding of the distribution of hydroxyapatite and calcium oxide, and Figure 14(3b) is the interior detail of the formed micropores.

### 3.6. Cytotoxicity Evaluation of Composite Scaffolds

Cell activity testing of medical devices implanted in humans is a fundamental step in verifying their biocompatibility. The cell activity test is performed by culturing cells in vitro and using the specimen as an indicator of the proliferation and differentiation attachment of cells to discern the biocompatibility of the specimen based on the cell growth. The steps of the cell activity test are:

(1) Stent pre-treatment

Each group of scaffolds to be tested was prepared at a weight of 1 g, and then placed under UV light for 5 h and set aside.

(2) Preparation of extracts

The test vials were sterilized and marked on a sterile operating table, and then the test vials were placed in the marked test vials with the extracts, and the test vials were extracted at a constant temperature of 37 °C for 30 h. After 30 h, the extracts were filtered and stored in a low-temperature freezer.

(3) Cell Processing

In this paper, c5.18 chondrocytes (purchased from Biotechnology Co., Ltd., Shanghai, China) were selected for the test. Chondrocytes were put into the culture bottle, and then the culture medium (DMEM medium, purchased from Sigma-Aldrich, Schnelldorf, Germany) was added. When a large number of chondrocytes grew along the bottle wall, the cell culture medium in the bottle was poured out and PBS solution (Phosphate-buffered saline, pH: 7.4, purchased from Sigma-Aldrich, Schnelldorf, Germany) was added. In order to promote cell wall detachment, 2 mL of trypsin (trypsin purchased from Sigma-Aldrich, Schnelldorf, Germany) with a 0.25% content was added, and then the dropper was used to absorb the liquid in the nutrient bottle to wash the bottle wall. When most of the chondrocytes fall off, transfer the culture liquid containing cells to the centrifuge tube, centrifuge for 5 min, remove the upper liquid, and add the culture liquid, and the cell suspension is successfully prepared.

(4) MTT (Methylthiazole) assay for cell activity

After the cells were morphologically normalized, the medium in the wells was aspirated by pipette, and 10 mL of MTT solution was added, and then the incubation was continued in a CO_2_ incubator. Four hr later, purple spots were observed when removed. The MTT solution was aspirated and 100 µL of DMSO (Dimethyl sulfoxide, purchased from Xiao You Biotechnology, Shanghai, China) was added. The solution was placed in an enzymatic standard for absorbance detection. The wavelength was set to 570 nm, and the average of five sets of specimens was taken at 20-h intervals.

**Figure 15:**
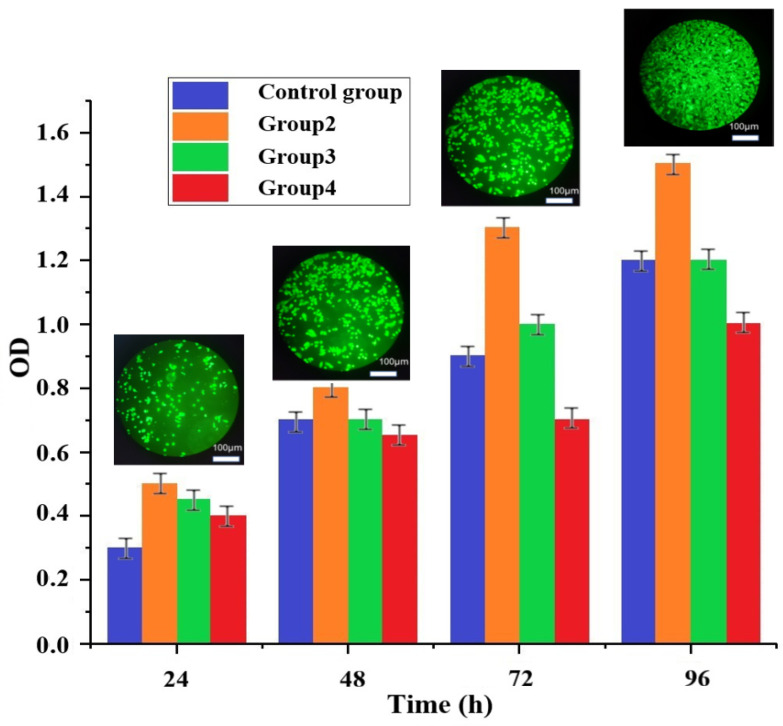
Cell activity observation graph (insert graph is group 2).

In order to verify the biocompatibility of the composite materials and the influence of the scaffold on cells, c5.18 chondrocytes were used for experimental research. From the data in Figure 15, it can be found that the OD (Optical Density) values of all groups increased with time, indicating that the cell growth condition was better in each group of the experiment. According to the changes in the cytotoxicity rating, it can be found that group 2 and group 3 maintained a cytotoxicity grade of 0 with time, while group 4 decreased to grade 1 after 48 h. Both cytotoxicity grade 0 and grade 1 can be considered qualified. Thus, although the cytotoxicity grade of the fourth group decreased, it was still within the permissible range of compliance. These phenomena also indicate that the increase of ZrO_2_ content, although improving the mechanical properties of the artificial bone structure, also sacrifices biocompatibility to some extent. The biocompatibility of the three rationing schemes was evaluated comprehensively by cytotoxicity grade evaluation experiments: group 2 (10% ZrO_2_, cytotoxicity grade 0) > group 3 (20% ZrO_2_, cytotoxicity grade 0) > group 4 (30% ZrO_2_, cytotoxicity grade 1). The samples was cultured in a CO_2_ constant temperature incubator (Shanghai Shenan Medical Equipment, Shanghai, China), After 96 h, the Cell Counting Kit-8 (CCK-8, Dojindo, Kumamoto, Japan) assay was used to count the cells and analyze cell viability. The four groups of cells have grown by more than 100%, and the small picture is the cell fluorescence picture of group 2, which was observed using a Fluorescent inverted microscope (Thermo Fisher, Waltham, MA, USA) and an Enzyme calibrator (Thermo Fisher, Waltham, MA, USA). Nie R. et al. [42] developed a personalized MXene composite hydrogel scaffold GelMA/β-TCP/sodium alginate (Sr2+)/MXene (Ti3C2) (GTAM) with photothermal antibacterial, which play synergistic roles in antibacterial and osteogenic effects. Compared with their results, our composite materials have a higher cell survival rate, which shows that they have better biocompatibility.

## 4. Conclusions

This paper focuses on three aspects: the selection of new composite materials, the preparation of composite materials considering the requirements of both mechanical properties and biological compatibility after implantation into the human body as artificial bone implants, and the verification that each component is non-toxic to the human body. The second aspect involved the study of the printing process. The composite material was extruded from the silo of the printing device and deposited onto the printing substrate by pressure through the injection molding technique, and the influencing factors of this process were theoretically studied and experimentally verified. The non-toxic performance of the composite in terms of cytotoxicity, with the following main conclusions.

1. The mechanical properties of the artificial bone made by different grouping ratio schemes under three structures were obtained. Depending on the percentage of ZrO2, compressive strengths of 5–17 MPa and elastic moduli of 52–174 MPa were obtained under the linear internal structure, and compressive strengths of 6–19 MPa and 12–25 MPa and elastic moduli of 64–196 MPa and 126–259 MPa were obtained by improving the internal structure to the folded and honeycomb types, respectively.
2. In vitro degradation experiments found that the surface of the scaffold can be a deposited apatite layer. The mechanical strength decreases with decomposition with the generation of an apatite layer and increase.
3. Experiments on the cytotoxicity of artificial bone scaffolds were conducted for three groups of rationing schemes, and the cytotoxicity of group 2 and group 3 showed a grade of 0, and group 4 showed a grade of 1. As the number of days of culture increases, the cells survive better and better. Because the porosity of the cartilage scaffold is high, and there are many holes on its micro surface, the existence of these holes provides more growth space for the growth of chondrocytes, which can better facilitate the material transfer between cells. High porosity also increases the surface area of the scaffold, providing more space for cell growth and proliferation. After 96 h, the four groups of cells have grown to more than 100%, which shows that the biomaterial has good biocompatibility.
4. The observed results showed that the cells grew in good condition on the scaffold surface.

In this work, some achievements have been made in the research of HA-ZrO_2_-PVA composite bone biological scaffolds, and the following work will be considered in the future work due to the current limitations of the experiment facilities:

(1) The sintering temperature and sintering process have an influence on the molding of artificial bone. In this study, the only sintering experiment was conducted at 1080 °C, so the influence of temperature on the performance of the sintered scaffold will be considered in the future work.
(2) There are few test items for biocompatibility. Due to the limitation of the actual conditions and equipment that available in our laboratory, not all test items can be carried out in our laboratory, and only three main test items can be evaluated. We could cooperate with hospital or medical research institutes to develop composite scaffolds for animal experiments to verify the biocompatibility of the composite material.

## Figures and Tables

**Table 1:** Composition of composite slurry with different ratios.

Number	HA (g)	ZrO_2_ (g)	PVA (g)
1	14.00	0.00	4.00
2	12.60	1.40	4.00
3	11.20	2.80	4.00
4	9.80	4.20	4.00

**Table 2:** Printed parameters of each composite group.

Group Name	Print Height (mm)	Nozzle Size (μm)	Movement (mm/s)	Pressure (bar)
1	20	400	10	1
				2
				3
				4
				5
2	20	400	10	1
				2
				3
				4
				5
3	20	400	10	1
				2
				3
				4
				5
4	20	400	10	1
				2
				3
				4
				5

## Data Availability

The study did not report any data.
